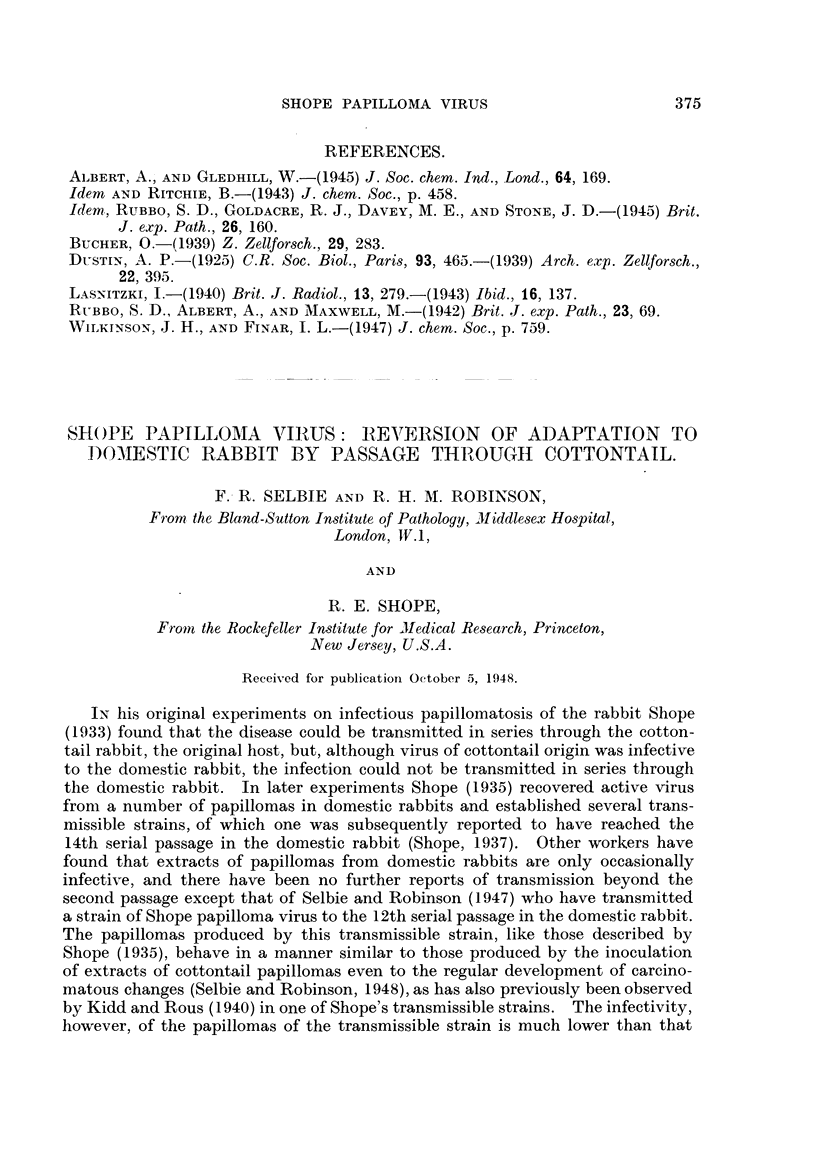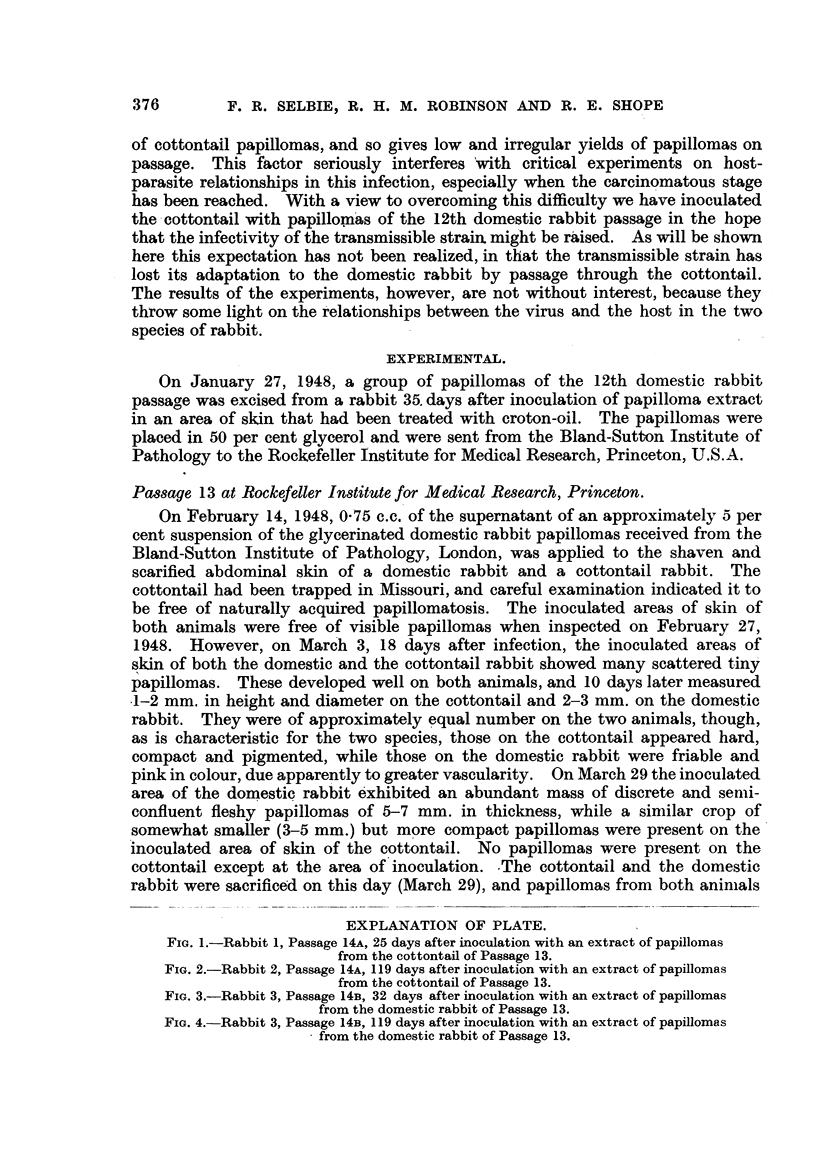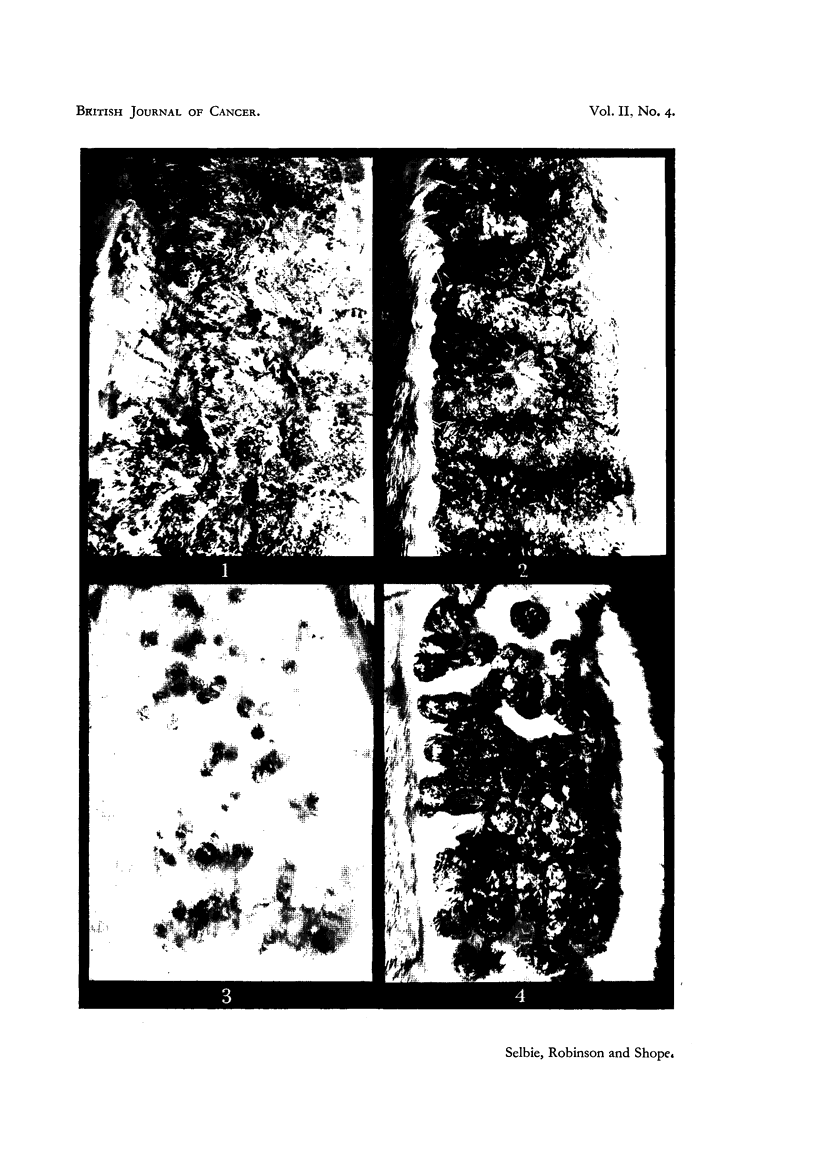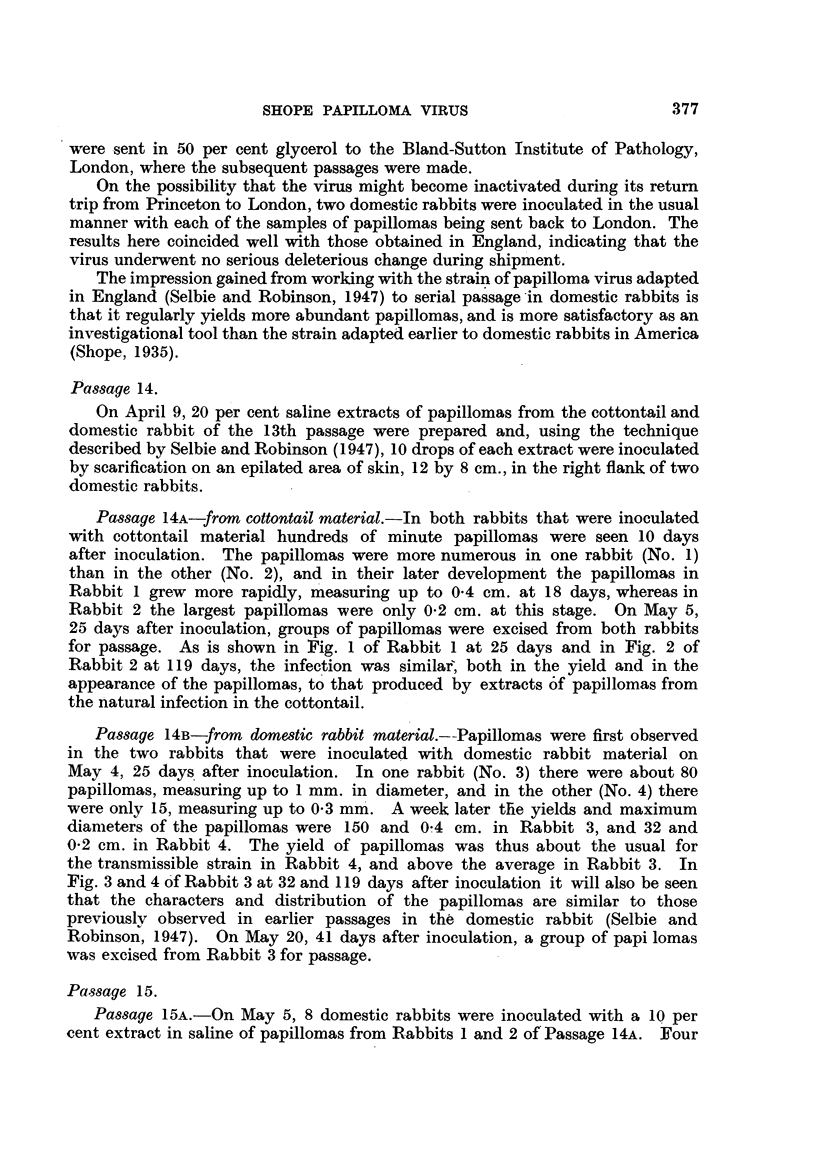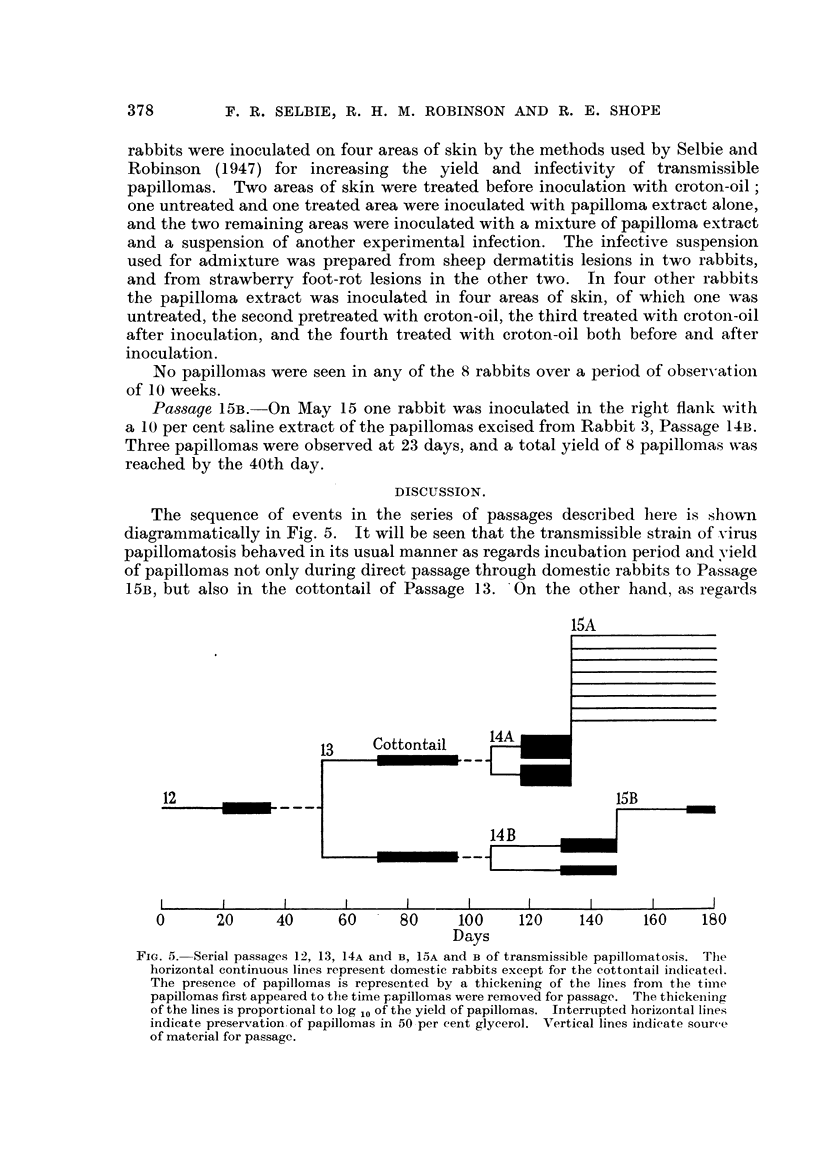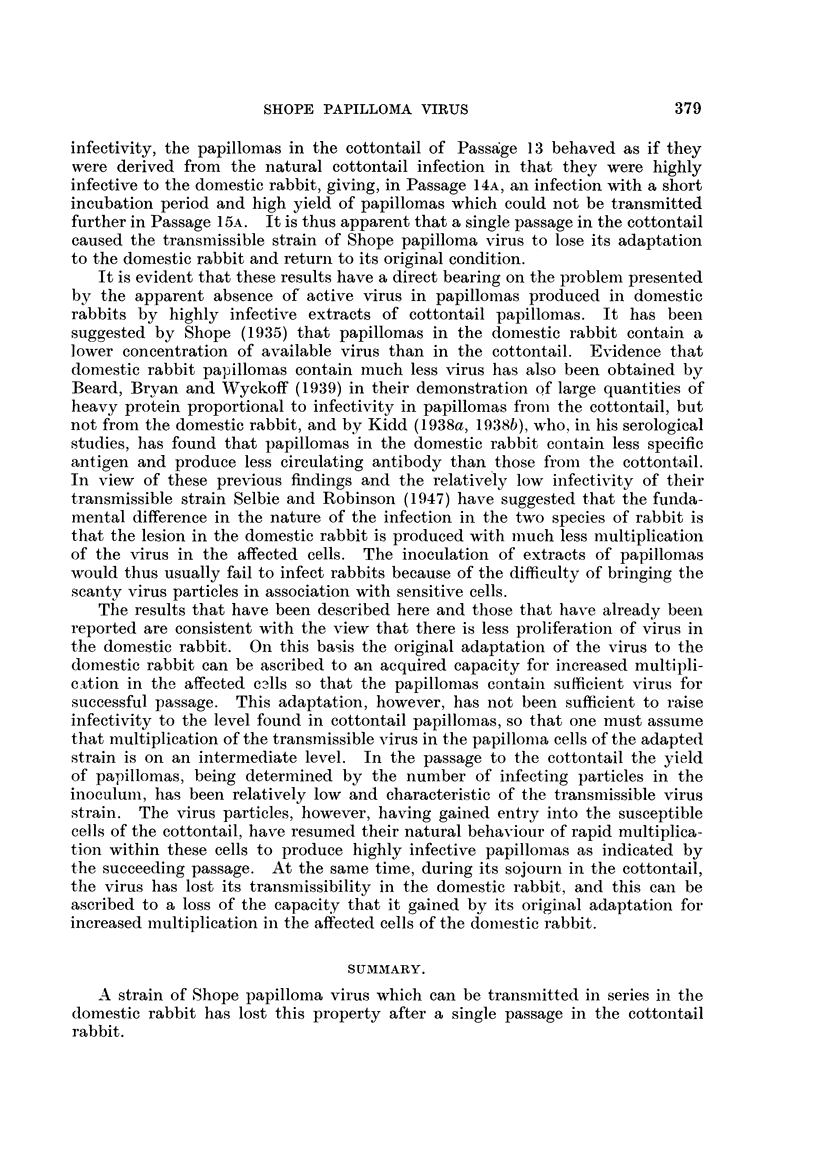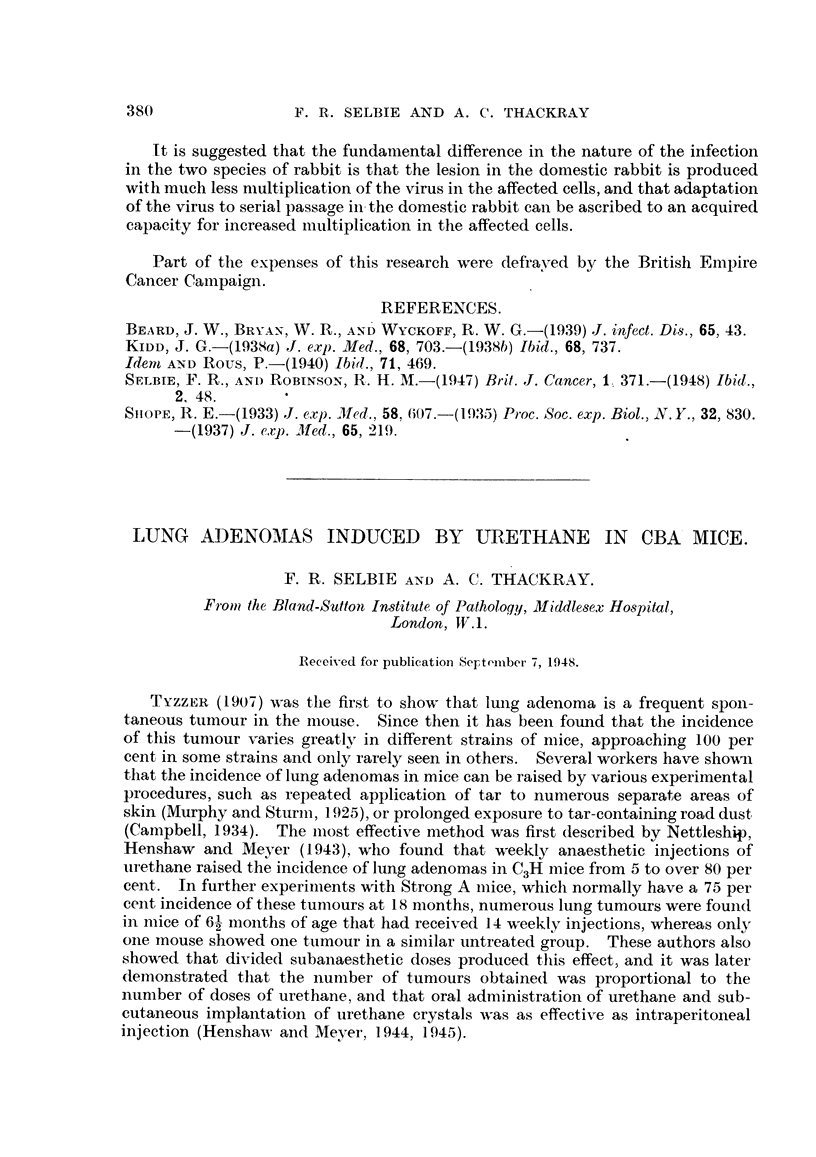# Shope Papilloma Virus: Reversion of Adaptation to Domestic Rabbit by Passage through Cottontail

**DOI:** 10.1038/bjc.1948.41

**Published:** 1948-12

**Authors:** F. R. Selbie, R. H. M. Robinson, R. E. Shope

## Abstract

**Images:**


### REFERENCES.

ALBERT, A., AND GLEDHILL, W.—(1945) *J. Soc. chem. Ind., Lond.*, **64**, 169.
*Idem* AND RITCHIE, B.—(1943) *J. chem. Soc.*, p. 458.
*Idem*, RUBBO, S. D., GOLDACRE, R. J., DAVEY, M. E., AND STONE, J. D.—(1945) *Brit. J. exp. Path.*, **26**, 160.
BUCHER, O.—(1939) *Z. Zellforsch.*, **29**, 283.
DUSTIN, A. P.—(1925) *C.R. Soc. Biol., Paris*, **93**, 465.—(1939) *Arch. exp. Zellforsch.*, **22**, 395.
LASNITZKI, I.—(1940) *Brit. J. Radiol.*, **13**, 279.—(1943) *Ibid.*, **16**, 137.
RUBBO, S. D., ALBERT, A., AND MAXWELL, M.—(1942) *Brit. J. exp. Path.*, **23**, 69.
WILKINSON, J. H., AND FINAR, I. L.—(1947) *J. chem. Soc.*, p. 759.

### SHOPE PAPILLOMA VIRUS: REVERSION OF ADAPTATION TO DOMESTIC RABBIT BY PASSAGE THROUGH COTTONTAIL.

F. R. SELBIE AND R. H. M. ROBINSON,

*From the Bland-Sutton Institute of Pathology, Middlesex Hospital, London, W.1,*

AND

R. E. SHOPE,

*From the Rockefeller Institute for Medical Research, Princeton, New Jersey, U.S.A.*

Received for publication October 5, 1948.

IN his original experiments on infectious papillomatosis of the rabbit Shope (1933) found that the disease could be transmitted in series through the cottontail rabbit, the original host, but, although virus of cottontail origin was infective to the domestic rabbit, the infection could not be transmitted in series through the domestic rabbit. In later experiments Shope (1935) recovered active virus from a number of papillomas in domestic rabbits and established several transmissible strains, of which one was subsequently reported to have reached the 14th serial passage in the domestic rabbit (Shope, 1937). Other workers have found that extracts of papillomas from domestic rabbits are only occasionally infective, and there have been no further reports of transmission beyond the second passage except that of Selbie and Robinson (1947) who have transmitted a strain of Shope papilloma virus to the 12th serial passage in the domestic rabbit. The papillomas produced by this transmissible strain, like those described by Shope (1935), behave in a manner similar to those produced by the inoculation of extracts of cottontail papillomas even to the regular development of carcinomatous changes (Selbie and Robinson, 1948), as has also previously been observed by Kidd and Rous (1940) in one of Shope's transmissible strains. The infectivity, however, of the papillomas of the transmissible strain is much lower than that of cottontail papillomas, and so gives low and irregular yields of papillomas on passage. This factor seriously interferes with critical experiments on host-parasite relationships in this infection, especially when the carcinomatous stage has been reached. With a view to overcoming this difficulty we have inoculated the cottontail with papillomas of the 12th domestic rabbit passage in the hope that the infectivity of the transmissible strain might be raised. As will be shown here this expectation has not been realized, in that the transmissible strain has lost its adaptation to the domestic rabbit by passage through the cottontail. The results of the experiments, however, are not without interest, because they throw some light on the relationships between the virus and the host in the two species of rabbit.

### EXPERIMENTAL.

On January 27, 1948, a group of papillomas of the 12th domestic rabbit passage was excised from a rabbit 35 days after inoculation of papilloma extract in an area of skin that had been treated with croton-oil. The papillomas were placed in 50 per cent glycerol and were sent from the Bland-Sutton Institute of Pathology to the Rockefeller Institute for Medical Research, Princeton, U.S.A.

*Passage* 13 *at Rockefeller Institute for Medical Research, Princeton.*

On February 14, 1948, 0·75 c.c. of the supernatant of an approximately 5 per cent suspension of the glycerinated domestic rabbit papillomas received from the Bland-Sutton Institute of Pathology, London, was applied to the shaven and scarified abdominal skin of a domestic rabbit and a cottontail rabbit. The cottontail had been trapped in Missouri, and careful examination indicated it to be free of naturally acquired papillomatosis. The inoculated areas of skin of both animals were free of visible papillomas when inspected on February 27, 1948. However, on March 3, 18 days after infection, the inoculated areas of skin of both the domestic and the cottontail rabbit showed many scattered tiny papillomas. These developed well on both animals, and 10 days later measured 1–2 mm. in height and diameter on the cottontail and 2–3 mm. on the domestic rabbit. They were of approximately equal number on the two animals, though, as is characteristic for the two species, those on the cottontail appeared hard, compact and pigmented, while those on the domestic rabbit were friable and pink in colour, due apparently to greater vascularity. On March 29 the inoculated area of the domestic rabbit exhibited an abundant mass of discrete and semi-confluent fleshy papillomas of 5–7 mm. in thickness, while a similar crop of somewhat smaller (3–5 mm.) but more compact papillomas were present on the inoculated area of skin of the cottontail. No papillomas were present on the cottontail except at the area of inoculation. The cottontail and the domestic rabbit were sacrificed on this day (March 29), and papillomas from both animals

EXPLANATION OF PLATE.

FIG. 1.—Rabbit 1, Passage 14A, 25 days after inoculation with an extract of papillomas from the cottontail of Passage 13.

FIG. 2.—Rabbit 2, Passage 14A, 119 days after inoculation with an extract of papillomas from the cottontail of Passage 13.

FIG. 3.—Rabbit 3, Passage 14B, 32 days after inoculation with an extract of papillomas from the domestic rabbit of Passage 13.

FIG. 4.—Rabbit 3, Passage 14B, 119 days after inoculation with an extract of papillomas from the domestic rabbit of Passage 13.

Selbie, Robinson and Shope.

were sent in 50 per cent glycerol to the Bland-Sutton Institute of Pathology, London, where the subsequent passages were made.

On the possibility that the virus might become inactivated during its return trip from Princeton to London, two domestic rabbits were inoculated in the usual manner with each of the samples of papillomas being sent back to London. The results here coincided well with those obtained in England, indicating that the virus underwent no serious deleterious change during shipment.

The impression gained from working with the strain of papilloma virus adapted in England (Selbie and Robinson, 1947) to serial passage in domestic rabbits is that it regularly yields more abundant papillomas, and is more satisfactory as an investigational tool than the strain adapted earlier to domestic rabbits in America (Shope, 1935).

*Passage* 14.

On April 9, 20 per cent saline extracts of papillomas from the cottontail and domestic rabbit of the 13th passage were prepared and, using the technique described by Selbie and Robinson (1947), 10 drops of each extract were inoculated by scarification on an epilated area of skin, 12 by 8 cm., in the right flank of two domestic rabbits.

*Passage* 14A—*from cottontail material.*—In both rabbits that were inoculated with cottontail material hundreds of minute papillomas were seen 10 days after inoculation. The papillomas were more numerous in one rabbit (No. 1) than in the other (No. 2), and in their later development the papillomas in Rabbit 1 grew more rapidly, measuring up to 0·4 cm. at 18 days, whereas in Rabbit 2 the largest papillomas were only 0·2 cm. at this stage. On May 5, 25 days after inoculation, groups of papillomas were excised from both rabbits for passage. As is shown in Fig. 1 of Rabbit 1 at 25 days and in Fig. 2 of Rabbit 2 at 119 days, the infection was similar, both in the yield and in the appearance of the papillomas, to that produced by extracts of papillomas from the natural infection in the cottontail.

*Passage* 14B—*from domestic rabbit material.*—Papillomas were first observed in the two rabbits that were inoculated with domestic rabbit material on May 4, 25 days after inoculation. In one rabbit (No. 3) there were about 80 papillomas, measuring up to 1 mm. in diameter, and in the other (No. 4) there were only 15, measuring up to 0·3 mm. A week later the yields and maximum diameters of the papillomas were 150 and 0·4 cm. in Rabbit 3, and 32 and 0·2 cm. in Rabbit 4. The yield of papillomas was thus about the usual for the transmissible strain in Rabbit 4, and above the average in Rabbit 3. In Fig. 3 and 4 of Rabbit 3 at 32 and 119 days after inoculation it will also be seen that the characters and distribution of the papillomas are similar to those previously observed in earlier passages in the domestic rabbit (Selbie and Robinson, 1947). On May 20, 41 days after inoculation, a group of papi lomas was excised from Rabbit 3 for passage.

*Passage* 15.

*Passage* 15A.—On May 5, 8 domestic rabbits were inoculated with a 10 per cent extract in saline of papillomas from Rabbits 1 and 2 of Passage 14A. Four rabbits were inoculated on four areas of skin by the methods used by Selbie and Robinson (1947) for increasing the yield and infectivity of transmissible papillomas. Two areas of skin were treated before inoculation with croton-oil; one untreated and one treated area were inoculated with papilloma extract alone, and the two remaining areas were inoculated with a mixture of papilloma extract and a suspension of another experimental infection. The infective suspension used for admixture was prepared from sheep dermatitis lesions in two rabbits, and from strawberry foot-rot lesions in the other two. In four other rabbits the papilloma extract was inoculated in four areas of skin, of which one was untreated, the second pretreated with croton-oil, the third treated with croton-oil after inoculation, and the fourth treated with croton-oil both before and after inoculation.

No papillomas were seen in any of the 8 rabbits over a period of observation of 10 weeks.

*Passage* 15B.—On May 15 one rabbit was inoculated in the right flank with a 10 per cent saline extract of the papillomas excised from Rabbit 3, Passage 14B. Three papillomas were observed at 23 days, and a total yield of 8 papillomas was reached by the 40th day.

DISCUSSION.

The sequence of events in the series of passages described here is shown diagrammatically in Fig. 5. It will be seen that the transmissible strain of virus papillomatosis behaved in its usual manner as regards incubation period and yield of papillomas not only during direct passage through domestic rabbits to Passage 15B, but also in the cottontail of Passage 13. On the other hand, as regards

FIG. 5.—Serial passages 12, 13, 14A and B, 15A and B of transmissible papillomatosis. The horizontal continuous lines represent domestic rabbits except for the cottontail indicated. The presence of papillomas is represented by a thickening of the lines from the time papillomas first appeared to the time papillomas were removed for passage. The thickening of the lines is proportional to log $_{10}$ of the yield of papillomas. Interrupted horizontal lines indicate preservation of papillomas in 50 per cent glycerol. Vertical lines indicate source of material for passage.

infectivity, the papillomas in the cottontail of Passage 13 behaved as if they were derived from the natural cottontail infection in that they were highly infective to the domestic rabbit, giving, in Passage 14A, an infection with a short incubation period and high yield of papillomas which could not be transmitted further in Passage 15A. It is thus apparent that a single passage in the cottontail caused the transmissible strain of Shope papilloma virus to lose its adaptation to the domestic rabbit and return to its original condition.

It is evident that these results have a direct bearing on the problem presented by the apparent absence of active virus in papillomas produced in domestic rabbits by highly infective extracts of cottontail papillomas. It has been suggested by Shope (1935) that papillomas in the domestic rabbit contain a lower concentration of available virus than in the cottontail. Evidence that domestic rabbit papillomas contain much less virus has also been obtained by Beard, Bryan and Wyckoff (1939) in their demonstration of large quantities of heavy protein proportional to infectivity in papillomas from the cottontail, but not from the domestic rabbit, and by Kidd (1938*a*, 1938*b*), who, in his serological studies, has found that papillomas in the domestic rabbit contain less specific antigen and produce less circulating antibody than those from the cottontail. In view of these previous findings and the relatively low infectivity of their transmissible strain Selbie and Robinson (1947) have suggested that the fundamental difference in the nature of the infection in the two species of rabbit is that the lesion in the domestic rabbit is produced with much less multiplication of the virus in the affected cells. The inoculation of extracts of papillomas would thus usually fail to infect rabbits because of the difficulty of bringing the scanty virus particles in association with sensitive cells.

The results that have been described here and those that have already been reported are consistent with the view that there is less proliferation of virus in the domestic rabbit. On this basis the original adaptation of the virus to the domestic rabbit can be ascribed to an acquired capacity for increased multiplication in the affected cells so that the papillomas contain sufficient virus for successful passage. This adaptation, however, has not been sufficient to raise infectivity to the level found in cottontail papillomas, so that one must assume that multiplication of the transmissible virus in the papilloma cells of the adapted strain is on an intermediate level. In the passage to the cottontail the yield of papillomas, being determined by the number of infecting particles in the inoculum, has been relatively low and characteristic of the transmissible virus strain. The virus particles, however, having gained entry into the susceptible cells of the cottontail, have resumed their natural behaviour of rapid multiplication within these cells to produce highly infective papillomas as indicated by the succeeding passage. At the same time, during its sojourn in the cottontail, the virus has lost its transmissibility in the domestic rabbit, and this can be ascribed to a loss of the capacity that it gained by its original adaptation for increased multiplication in the affected cells of the domestic rabbit.

### SUMMARY.

A strain of Shope papilloma virus which can be transmitted in series in the domestic rabbit has lost this property after a single passage in the cottontail rabbit.

It is suggested that the fundamental difference in the nature of the infection in the two species of rabbit is that the lesion in the domestic rabbit is produced with much less multiplication of the virus in the affected cells, and that adaptation of the virus to serial passage in the domestic rabbit can be ascribed to an acquired capacity for increased multiplication in the affected cells.

Part of the expenses of this research were defrayed by the British Empire Cancer Campaign.

REFERENCES.

BEARD, J. W., BRYAN, W. R., AND WYCKOFF, R. W. G.—(1939) *J. infect. Dis.*, **65**, 43.
KIDD, J. G.—(1938*a*) *J. exp. Med.*, **68**, 703.—(1938*b*) *Ibid.*, **68**, 737.
*Idem* AND ROUS, P.—(1940) *Ibid.*, **71**, 469.
SELBIE, F. R., AND ROBINSON, R. H. M.—(1947) *Brit. J. Cancer*, **1**, 371.—(1948) *Ibid.*, **2**, 48.
SHOPE, R. E.—(1933) *J. exp. Med.*, **58**, 607.—(1935) *Proc. Soc. exp. Biol., N.Y.*, **32**, 830. —(1937) *J. exp. Med.*, **65**, 219.

---

### LUNG ADENOMAS INDUCED BY URETHANE IN CBA MICE.

F. R. SELBIE AND A. C. THACKRAY.